# Variations in the Peritrophic Matrix Composition of Heparan Sulphate from the Tsetse Fly, *Glossina morsitans morsitans*

**DOI:** 10.3390/pathogens7010032

**Published:** 2018-03-19

**Authors:** Evelyn Rogerson, Julien Pelletier, Alvaro Acosta-Serrano, Clair Rose, Sarah Taylor, Scott Guimond, Marcelo Lima, Mark Skidmore, Edwin Yates

**Affiliations:** 1 Liverpool School of Tropical Medicine, Pembroke Place, Liverpool L3 5QA, UK; eayates@liv.ac.uk (E.R.); alvaro.acosta-serrano@lstmed.ac.uk (A.A.-S.); Clair.Rose@lstmed.ac.uk (C.R.); 2 Molecular & Structural Bioscience, School of Life Sciences, Keele University, Keele ST5 5BG, UK; j.pelletier@keele.ac.uk; 3 Institute of Integrative Biology, University of Liverpool, Liverpool L69 7ZB, UK; Sarah.Taylor@liverpool.ac.uk (S.T.); S.Guimond@liverpool.ac.uk (S.G.); 4 Department of Biochemistry, Universidade Federal de São Paulo, São Paulo 04044-020, Brazil; mlimagb@gmail.com

**Keywords:** *Glossina morsitans morsitans*, tsetse fly, *Trypanosoma brucei*, heparan sulphate, peritrophic matrix

## Abstract

Tsetse flies are the principal insect vectors of African *trypanosomes*—sleeping sickness in humans and Nagana in cattle. One of the tsetse fly species, *Glossina morsitans morsitans*, is host to the parasite, *Trypanosoma brucei*, a major cause of African trypanosomiasis. Precise details of the life cycle have yet to be established, but the parasite life cycle involves crossing the insect peritrophic matrix (PM). The PM consists of the polysaccharide chitin, several hundred proteins, and both glycosamino- and galactosaminoglycan (GAG) polysaccharides. Owing to the technical challenges of detecting small amounts of GAG polysaccharides, their conclusive identification and composition have not been possible until now. Following removal of PMs from the insects and the application of heparinases (bacterial lyase enzymes that are specific for heparan sulphate (HS) GAG polysaccharides), dot blots with a HS-specific antibody showed heparan sulphate proteoglycans (HSPGs) to be present, consistent with *Glossina morsitans morsitans* genome analysis, as well as the likely expression of the HSPGs syndecan and perlecan. Exhaustive HS digestion with heparinases, fluorescent labeling of the resulting disaccharides with BODIPY fluorophore, and separation by strong anion exchange chromatography then demonstrated the presence of HS for the first time and provided the disaccharide composition. There were no significant differences in the type of disaccharide species present between genders or between ages (24 vs*.* 48 h post emergence), although the HS from female flies was more heavily sulphated overall. Significant differences, which may relate to differences in infection between genders or ages, were evident, however, in overall levels of 2-*O*-sulphation between sexes and, for females, between 24 and 48 h post-emergence, implying a change in expression or activity for the 2-*O*-sulphotransferase enzyme. The presence of significant quantities of disaccharides containing the monosaccharide GlcNAc6S contrasts with previous findings in *Drosophila melanogaster* and suggests subtle differences in HS fine structure between species of the *Diptera*.

## 1. Introduction

The tsetse fly, *Glossina morsitans morsitans* (*Glossina m. m.*), a member of the order *Diptera*, is one of seven savannah flies in the morsitans group, which inhabit West, Central, and East Africa. It is the principal insect vector of African trypanosomiasis (sleeping sickness in humans and Nagana in cattle) caused mainly by the protozoan *Trypanosoma brucei* [1]. In contrast to other *Diptera* that carry parasites, such as mosquitoes, both male and female tsetse are obligate blood-feeders. 

Following ingestion of a blood meal containing *trypanosomes* by the insect, the parasite undergoes a complex series of developmental stages before transmission back to the vertebrate host. *Trypanosomes* differentiate and multiply in the insect midgut and then migrate to the salivary glands, where they undergo a further round of differentiation into epimastigotes, proliferate and differentiate to form infective metacyclic trypomastigotes, and are then transferred to a new host when a blood meal is taken [2]. Little is known at the molecular level regarding the basis for the recognition of the insect tissue by the parasite, or of the macromolecules with which the parasite comes into contact in the fly. A key part of the parasite lifecycle, the procyclic trypomastigote stage, occurs within the ectoperitrophic space in contact with the peritrophic matrix (PM). 

Here, we aim to identify unambiguously one of the major components of the PM of *Glossina m. m.*, heparan sulphate (HS), together with variations in its structure 24 and 48 h after emergence. This will also identify potential differences between ages and genders, to enable future understanding of the interactions of the parasite and symbiotic bacteria with the PM. 

### 1.1. The Peritrophic Matrix of Glossina morsitans morsitans

The acellular PM, which is in *Glossina m. m.*, is formed by continual secretion by specialised cells and constitutes a physical barrier to enzymes, digested material, and ingested toxins. The PM is just over 300 nm thick and formed of three principal layers [3] comprising the polysaccharide chitin and over 300 proteins [4], as well as other polysaccharides, which are proposed to be of both the glycosaminoglycan and galactosaminoglycan (GAG) types [5].

### 1.2. The Composition of the Peritrophic Matrix May Influence Infection and Tissue Tropism

The number of blood meals taken, the immune status of the insect [6] and host defense peptides [7], and the redox state of the gut lumen have all been linked to transmission of the pathogen to the insect vector; less oxidizing conditions correspond to higher susceptibility to infection. Furthermore, a thin PM as found in unfed flies, or a damaged PM such as that resulting from the disruption of peritrophin production, also lead to higher infection rates in the flies [8,9,10]. Thus, it is feasible that the status of the PM may be a significant determinant of infection by the parasite and the composition of its constituents, and any variations with age and gender are of potential significance to the infection process. 

Owing to the technical challenges involved in extracting and detecting small amounts of material from the organs of flies, little is known currently concerning the detailed composition of the putative GAG components in many insects, and especially the HS composition of their individual organs. The corresponding genes that have been identified in the tsetse fly genome include a galactosyltransferase for initiation of GAG chain biosynthesis and a number of sulphotransferase superfamily members [4,11]. These findings suggest that GAGs form one component of the PM, but unequivocal detection of GAGs employing direct biochemical means has yet to be reported in *Glossina.*

It is conceivable that both the chitin and GAGs of the PM serve as a means of recognition by the parasite, and it is known that another parasite, *T. cruzi*, invades mammalian host cardiomyocytes via attachment to host cell heparan sulphate (HS) but not to chondroitin sulphate (CS) [12]. Glycosaminoglycans in particular may play a similar role in the PM of *Glossina m. m.* and facilitate invasion by *T. brucei,* since GAGs are established ligands in mammalian hosts for a range of bacteria [13]. In addition, GAGs are expressed on almost all cells but are also variable in structure; hence, could serve as one source of tissue tropism. An additional role for GAGs may relate to the adhesion of commensal bacteria, such as the symbiont *Sodalis glossinidius*, with which the PM is in close contact and which has been linked to susceptibility to infection by *trypanosomes*. This bacterium, which possesses a chitinase and whose principal carbon source is *N*-acetyl d-glucosamine (the repeating unit of chitin and also present in HS), is transmitted through secretions from maternal milk gland secretions [14], facilitating vertical transmission. Furthermore, many proteins from *S. glossinidius* have been identified in the PM of the tsetse fly, supporting a close association. 

The likelihood of insects becoming infected with *trypanosomes* diminishes with their age [15], while starvation is associated with increased susceptibility to infection. An explanation of the variation of infectivity with distinct developmental or feeding states may lie in differences in the thickness and structure of the PM, including their GAG composition and variations between genders and over time. Any difference could potentially result in altered recognition and adhesion by the parasite, or it could alter the adhesion or status of symbiotic bacteria, with secondary effects on infection. 

### 1.3. Proteoglycans in Other Members of the Diptera

Glycosaminoglycan-bearing HSPGs are highly conserved across Dipteran insects, in which they are involved in important stages of early development [16]. These HSPGs include transmembrane syndican [17], GPI-anchored glypican, and secreted aggrecan and perlecan. Some of these proteoglycans can bear polysaccharide chains of the HS or CS type and could be involved in disease processes, either as a means of attachment of parasites, or commensal bacteria, or in some more indirect capacity. 

It is, therefore, important to define in more detail the molecular composition of the components of the PM. Here, the presence of HS in the PM of *Glossina m. m.* is demonstrated by biochemical means. Details of its composition in terms of the disaccharide content, as well as the individual position of the substitution, are reported, together with comparisons between unfed (to avoid any possibility of contamination with HS from the blood meal) male and female flies at 24 and 48 h post emergence.

### 1.4. The Overall Structure of Heparan Sulphate Polysaccharides

Heparan sulphate is a member of the GAG class of polysaccharides, whose structure is known to vary between species and between tissue and cell types within individuals. In broad terms, HS is composed of linear chains of 1–4 linked disaccharide repeating units (Figure 3A), themselves comprising an uronic acid (either β d-GlcA or α l-IdoA, that can be 2-*O*-sulphated) and an α d-glucosamine residue, which can be 6-*O*-sulphated, *N*-sulphated, or *N*-acetylated. In mammalian HS, there is an overall domain structure; longer stretches that are rich in the repeating disaccharide, d-GlcA α (1–4) d-GlcNAc, containing low levels of sulphation, are interspersed with shorter but more highly sulphated regions, which are thought to be the principal sites of interaction with proteins. 

## 2. Results

### 2.1. Heparan Sulphate Proteoglycans Exist in the Peritrophic Matrix of Glossina morsitans morsitans

**Figure 1:**
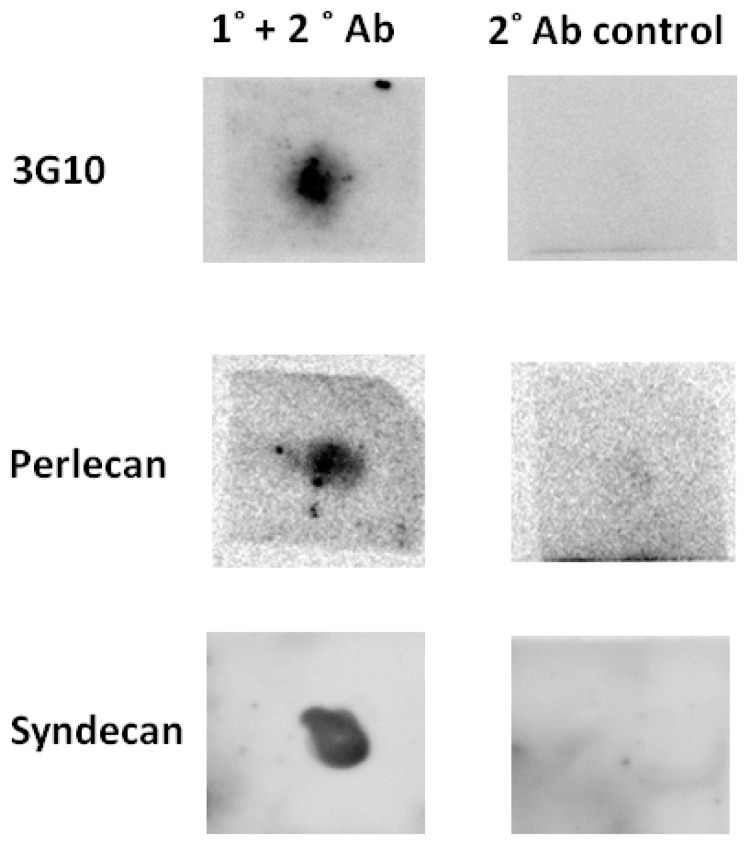
Dot blots of extracted PM material from *Glossina m. m*. using: 3G10 antibody (**upper left**), specific against the stub that is exposed in HS following heparinase treatment and considered definitive evidence of heparan sulphate proteoglycans. Antibody specific against human, rat, mouse, cow, pig, and fish perlecan; (**middle left**). Antibody specific against syndecan-3 in human, rat, and mouse (**lower left**). Controls—secondary antibody without primary: donkey anti-mouse (**top and middle right**); donkey anti-goat (**lower right**).

The presence of HSPGs from the PM of *Glossina m. m.* was demonstrated by dot blot, through binding of the 3G10 monoclonal antibody, which recognises specifically the newly-formed non-reducing end ‘stub’ following digestion (Figure 1 (upper)) of the HS chains of HSPGs with heparinase enzymes (here, heparinase III EC 4.2.2.8) [18,19] that cuts less sulphated regions of HS. The probable presence of perlecan and syndecan (Figure 1 (middle and lower left) was also established by binding of polyclonal Syndecan-3 (Sdc3), recognizing *N*-terminus peptide of Sdc3, and monoclonal perlecan (Plc) antibodies recognizing the IV domain of Plc (manufacturers’ data sheets), although the identifications of particular HSPG core proteins must remain tentative, because these antibodies were not raised against proteins from *Glossina*. In the case of the anti-syndecan antibody, documented cross-reactivity is with mammalian and piscine syndecan, while for the anti-perlecan antibody, it is with rat, mouse, and human forms.

### 2.2. It Is Likely That Only One Heparan Sulphate Proteoglycan Exists in Glossina morsitans morsitans

**Figure 2:**
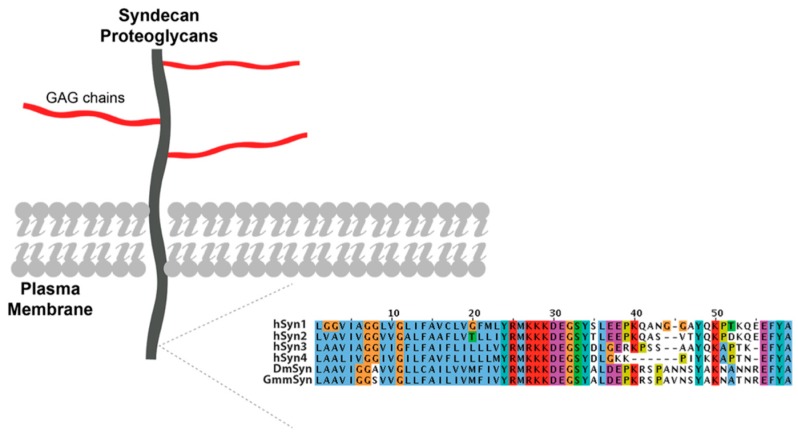
Schematic of syndecan proteoglycan and (inset) *N*-terminus sequence alignment of 4 human syndecans (hsynl to 4), *Drosophila* syndecan (DmSyn), and putative syndecan from *Glossina morsitans morsitans* (GmmSyn).

Using readily available human protein sequences (available at Uniprot), the presence of HSPGs and enzymes for HS biosynthesis within the genome sequence of *Glossina m. m.* was identified (Table 1). A single hit was found with significant similarity for the cytosolic domain of mammalian syndecan (Syndecan-1), shown schematically with partial amino acid sequences in Figure 2. All four mammalian proteins (Syndecans-1 to -4) match with one contig sequence, which may indicate that *Glossina m. m.* contains only one HSPG gene.

### 2.3. The Peritrophic Matrix of Glossina morsitans morsitans Contains Heparan Sulphate

The first step in the analysis of HS in the tsetse (and proof of its presence) is to digest exhaustively the polysaccharide with heparinase enzymes (heparinase I, II and III), a group of bacterial lyases from *Flavobacterium heparinium* that are specific for HS. This yields the constituent disaccharides, and these are identified by strong anion exchange chromatography (HPAEC) by reference to *bona fide* disaccharide standards. The relatively low sensitivity of these underivatised standards to detection, common to carbohydrate structures, necessitates the labeling of the digestion products and their subsequent high-sensitivity detection by fluorescence [20], and it is the difficulty of detecting such structures that has hindered their study in insect organs until now. This procedure enables the detection of HS-derived disaccharides from a reasonable number of PMs.

**Figure 3:**
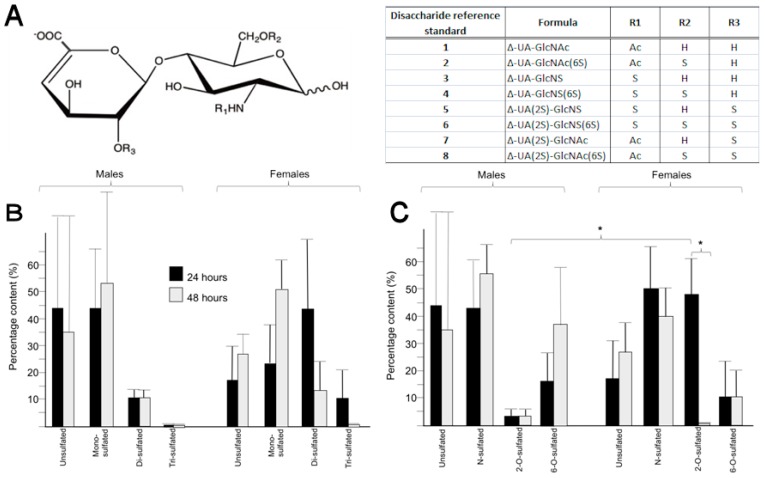
(**A**) Structures of the 8 unsaturated disaccharide standards derived from HS following exhaustive digestion with heparinases I, II, and III. GlcNAc; *N*-acetyl-d-glucosamine, GlcNS; *N*-sulphamino-d-glucosamine, 2S; 2-*O*-sulphate, 6S; 6-*O*-sulphate, Ac; COCH_3_, S; SO_3_^−^. H: Hydrogen (H). R1 to R3 refers to the appended functional groups at positions -2, -6 of GlcN residues and position -2 of IdoA, respectively; (**B**) Comparison of mono- (disaccharides 2, 3, and 7), di- (disaccharide 4, 5, and 8), and tri-sulphated disaccharide (disaccharide 6) in HS from 30 pooled peritrophic matrices from *Glossina m. m.* at 24 and 48 h. Error bars represent standard deviations of three technical repeats. Comparisons between proportions were made using z-statistics. There are no clear differences, in terms of the individual disaccharides, between male and female or between 24 and 48 h; (**C**) Comparison of *N*- (disaccharides 3, 4, 5, and 6), 2-*O*- (disaccharides 5, 6, 7, and 8), and 6-*O*- (disaccharides 2, 4, 6, and 8), sulphated disaccharides from 30 pooled peritrophic matrices of *Glossina m. m.* from males and females at 24 and 48 h. Error bars represent standard deviations for three technical repeats. There are significant differences (*p* < 0.05, indicated by *) between the levels of 2-*O*-sulphation in males and females at 48 h and for females between 24 and 48 h.

Susceptibility to lysis by the bacterial lyase enzymes, heparinase I, II, and III (originally from *Flavobacterium heparinum*), to generate disaccharides 1–8 (Figure 3), which, following labeling with the fluorophore BODIPY, elute on HPAEC under a linear salt gradient and their identification with reference to known standards, is proof of the presence of HS [21]. Thus, detection of the disaccharides reported in Figure 3B,C allows HS to be identified unambiguously as a component of the PM of *Glossina m. m.*

### 2.4. Heparan Sulphate from the Peritrophic Matrix Is Characterised by Moderate Levels of Sulphation

The HS from the PM is characterised by moderate levels of overall sulphation, there being proportionally much lower levels of tri-sulphated disaccharide (range 1–11%) than other forms (range 11–53%) (Figure 3B). Taking averages across females and males, at both 24 and 48 h, provides an overall value of 0.95 sulphates per disaccharide (calculated from disaccharide composition by HPAEC) that is similar to mammalian HS [22], despite their evolutionary distance, but may be of interest, since many pathogens interact with HS in both insect and mammalian hosts. 

### 2.5. Differences in the Position of Sulphate Group Substitution, Particularly 2-O-Sulphation, Occur between Sexes and over Time

There were considerable variations in the proportion of individual disaccharides observed between sexes and between ages (determined 24 and 48 h post emergence) among the pools of PMs tested, which did not provide any statistically significant differences (Figure 3B). More interestingly, differences were observed when the positions of sulphation (which could imply involvement of particular HS-biosynthetic enzymes) within the disaccharides produced by digestion (N-S, 2-*O*-S or 6-*O*-S) were assessed, rather than the identity of the individual disaccharides comprising the polysaccharide (Figure 3C). Female flies at 24 h contained much higher levels of 2-*O*-sulphated disaccharides than at 48 h (females, 24 h: 48% vs. females, 48 h: 2%, *p* < 0.05), while the levels of 2-*O*-sulphated disaccharides in males was low throughout (Figure 3C). The content of 2-*O*-sulphated disaccharides between males and females at 48 h was also different (males, 48 h: 3% vs. females, 48 h: 48%, *p* < 0.05), and this may indicate differences in 2-*O*-sulphotransferase expression or activity, the de-*N*-acetylation and *N*-sulphation by the *N*-deacetylase/*N*-sulphotransferase enzymes (NDSTs), and/or epimerization of D-GlcA to L-IdoA by the C-5 epimerase enzyme (Epi), which is the enzymes that precedes 2-*O*-sulphation in HS biosynthesis. In contrast to *Drosophila* [23], in the other member of the *Diptera,* for which detailed studies of HS composition have been made, disaccharides containing GlcNAc6S (disaccharides 2 and 8) were apparent.

## 3. Discussion

The results establish that the PMs of both male and female *Glossina m. m* contain HS at 24 h and 48 h post-emergence. Distinct levels of 2-*O*-sulphation have been shown to be present in the HS of the PM of *Glossina m. m.*, particularly between genders and for females between 24 and 48 h. It is not known yet what effect, if any, this has on the infection or transmission rate of the parasite, and this could form the basis of future investigations. It is feasible that the ability of parasites and/or commensal bacteria to interact with the PM may be altered. For instance, the recognition and/or attachment of parasites, or commensal bacteria, may depend on the overall levels of sulphation and/or specific sequences in the GAGs. Furthermore, the change in expression over time (24 vs. 48 h) may be a source of tissue tropism and/or influence the timing of interactions between parasite or bacteria and host. A probable mechanism for any such differences is likely to originate in distinct protein binding characteristics of the various forms of HS and/or to the HSPGs to which they are attached, and this will form the basis of future investigations. 

Genome sequences corresponding to HS biosynthetic enzymes (EXT, NDST-1, 2OST-1, 6OST-1, and 3OST-1) were retrieved from the *Glossina m. m.* genome (Table 1), which suggested the presence of HSPGs and, together with the dot blot results (Figure 1), provided evidence for syndecan-like proteoglycans (Figure 2, Table 1) of significant sequence similarity with already identified proteins. The presence of HSPG in the PM, demonstrated by the 3G10 antibody result (Figure 1), was confirmed by the identification of HS disaccharides (Figure 3B,C). The most commonly observed HS disaccharides in the PM of *Glossina m. m.* were those that were the least sulphated, bearing no sulphates (disaccharide 1) or one sulphate (disaccharides 2, 3, and 7) (Figure 3B) and were consistent with the typical composition of HS, which is conserved throughout the animal kingdom (typically comprising disaccharides 1 and 3 in mammals, for instance). At the level of the identity of the HS disaccharides detected, there were no significant differences between sexes or ages (Figure 3B); however, the HS from female flies was more sulphated overall than that from males (Figure 3B,C), with ca. 1.2 sulphates per disaccharide compared to ca. 0.7 (calculated from populations of disaccharides obtained by HPAEC). The presence of HS in the PM of the fly implies that the full set of enzymes required for its biosynthesis is present in the *Glossina m. m.* genome. Beyond tentative identification of GAGs in the PM of *Glossina m. m.* [3,5], very little information regarding GAG polysaccharides amongst the *Diptera* has been reported until now, largely because of the technical challenges associated with extracting and detecting such small amounts of material. Undoubtedly, the best studied member of the *Diptera*, although not closely related to *Glossina*, is the model organism *Drosophila melanogaster,* for which homologues of the mammalian biosynthetic enzymes for HS have been found [23]. The detection of disaccharides containing the monosaccharide residue GlcNAc6S (as a component of the observed disaccharides 2 and 8) in *Drosophila* suggests that subtle differences in the HS biosynthetic machinery, or its regulation, exist between *Drosophila melanogaster* [23] and *Glossina m. m*. *Glossina* are thought to have arisen at least 40 million years ago [24], although an earlier origin has also been suggested [25], and, given the evolutionary distance of *Glossina* from *Drosophila*, comparisons must be viewed with circumspection. 

It is intriguing that, in mammalian (Chinese Hamster Ovary) cells, 2-*O*-sulphation of HS has been linked to the turnover of the polysaccharide [26], and it is conceivable that the observed differences in 2-*O*-sulphation, via the 2-*O*-sulphotransferase (2OST; of which only one form has been identified in biology) expression or activity levels, may indicate different rates of HS turnover between sexes in *Glossina*, which could also have implications for infectivity. The differences in HS structure evident between 24 and 48 h post-emergence confirm that HSPG turnover is faster than 24 h, which is in keeping with findings in other taxa and previous results governing proteoglycan turnover in the salivary glands of mosquitoes, also members of the *Diptera* [27].

The results of this investigation establish that HS is one of the GAGs present in the peritrophic matrix of *Glossina m. m.* and facilitate future studies of their role in attachment and infection by both parasites and commensal bacteria, which will shed further light on their complex life cycles. The comparisons of HS disaccharide composition have revealed differences in the proportion of sulphation at specific positions of the constituent disaccharides that comprise the HS polysaccharide chain, involving, in particular, 2-*O*-sulphation, which may be of relevance to the normal life-cycle in tsetse flies, including colonisation by bacteria, as well as the disease process and interactions with *trypanosomes*. 

## 4. Materials and Methods

### 4.1. Cultivation of Tsetse Fly (Glossina morsitans morsitans)

The colony of *Glossina morsitans morsitans* housed in the Liverpool School of Tropical Medicine (LSTM) was collected originally in Zimbabwe. The flies were maintained in a stable environment at an ambient temperature of 26 ± 2 °C and at humidity levels in the range 75–82%. Between 50 and 80 flies were kept, according to gender, in open-ended pots with mesh coverings. Newly emerged flies of both sexes were assigned for analysis of PM GAG composition and were dissected at 24 and 48 h without having taken a blood meal in order to avoid contamination with HS from blood. 

### 4.2. Isolation of Peritrophic Matrices

Thirty PMs were taken for each experimental set, and each PM was removed by dissection in PBS solution under a dissecting microscope, and small incisions were made on the anterior abdomen to facilitate its removal. The midgut was extracted and severed carefully to reveal the PM. The presence of the secondary symbiont *Sodalis glossinidius* was evident in the PMs. Excised PMs were then pooled in a micro-centrifuge tube containing 500 μL PBS. The tubes were centrifuged at 14,000× *g* for 10 min to ensure that all PM material had settled and the excess PBS was removed using a micropipette. The PMs were then washed (×3) in water to remove any excess salts before centrifugation at 14,000× *g* for 10 min, after which the water was removed and samples frozen in liquid nitrogen and stored at −80 °C until use.

### 4.3. Heparinase Treatment of Peritrophic Matrices

For the purposes of confirmation of the presence of HSPGs, 3G10, syndecan 3 (Sdc3), and perlecan (Pln) antibody recognition was achieved by dot blot. 30 pooled PMs (from male, unfed flies (24 and 48 h post-emergence frozen in PBS) were combined, defrosted on ice, and macerated by mechanical mixing. This mixture was precipitated overnight at −20 °C with 1.0 mL of methanol, followed by spinning and resuspension of the pellet in −20 °C acetone (0.5 mL); the supernatant was discarded and the pellet was retained. The precipitate was then re-suspended in 50 µL lyase buffer (25 mM sodium acetate, 5 mM calcium acetate, pH 7) and heparinase III enzyme (EC 4.2.2.8, 1.25 mIU, Ibex Technologies Inc., Mont-Royal, QC, Canada) added and incubated at 37 °C for 2 h before a further addition of the same amount of enzyme and an additional 2 h incubation. 

### 4.4. Dot Blots of Heparinase-Digested Peritrophic Matrices

20 µL (Sdc3)/30 µL (3G10/Plc) of the heparinase III-digested HSPG sample (from above, Section 4.3) was then applied to a nitrocellulose membrane (GE Healthcare, Little Chalfont, UK), dried, and blocked for an hour at room temperature with 5% (*w*/*v*) skimmed milk-TBST. The addition of polyclonal Sdc3 cross reacting with rat/mouse/human (R&D Systems, Abingdon, UK), monoclonal 3G10 (Amsbio, Abingdon, UK), and monoclonal Pln cross reacting with human/rat/mouse/cow/pig/fish (Abcam, Cambridge, UK) primary antibodies (1:1000 *v*/*v*) in 5% (*w*/*v*) milk-TBST were incubated overnight at 4 °C. Secondary donkey anti-mouse (Plc/3G10—Santa Cruz, Dallas, TX, USA) and donkey anti-goat (Sdc3- Santa Cruz, Dallas, TX, USA) were added 1:10,000 (*v*/*v*) in 5% (*w*/*v*) milk-TBST for an hour at room temperature. Blots were developed by washing the membrane twice in 500 µL TBST, followed by one TBS wash. These were incubated with ECL (R&D Systems, Abingdon, UK), 5 min and imaged using a QuantLaser (GE Healthcare, Little Chalfont, UK). Controls in which primary antibodies were absent returned blots with no signals, as expected (shown in Figure 1). 

For the analysis of HS disaccharides (Section 4.6 below), the peritrophic matrices were defrosted and digested for 24 h at 37 °C in 10 μL bacterial lyase enzymes comprising: 5 mIU of heparinase I (EC 4.2.2.7), II and III (EC 4.2.2.8) (IBEX Technologies Inc., Mont-Royal, QC, Canada) in lyase buffer (25 mM sodium acetate, 5 mM calcium acetate, pH 7). Following digestion, debris were removed using a 0.2 μm spin filter and centrifuged at 9.3× *g*, 5 min, repeated thrice, and the filter washed to ensure all material had been recovered. Samples were then frozen and lyophilised. 

### 4.5. In Silico Sequence Analysis

Known human protein sequences were used as templates for the search (Uniprot accession numbers shown in Table 1). Tblastn analyses [28] were performed on VectorBase website engine [29]. Protein sequence alignment was performed using the built-in alignment plugin available at The Universal Protein Resource (UniProt).

### 4.6. Fluorescent Labeling

The dried samples, containing putative HS disaccharides were labeled with 4-difluoro-7, 7-dimethyl-4-bora-3a, 4a-diaza-s-indeciene-3-propionohydrazide (BODIPY FL hydrazide, ThermoFisher Scientific, Loughborough, UK), forming a Schiff’s base (imine) with the reducing-end aldehyde of the disaccharides, which was then reduced using sodium borohydride to the more stable amine, providing substantially enhanced sensitivity suitable for HPAEC with fluorescence detection [20]. Labeling was carried out as follows: Briefly, 10 μL of a solution of BODIPY hydrazide in methanol (5 mg/mL) was added to each dry disaccharide sample prior to centrifugal evaporation. When dry, glacial acetic acid (10 μL 18% *v*/*v*, in DMSO) was added before vortexing. Samples were incubated in pigmented tubes for 4 h at R.T. in darkness, after which 10 μL of a 1 mM sodium borohydride solution was added and incubated for 30 min to reduce the imine. Samples were frozen in liquid nitrogen and lyophilised prior to analysis. Results are the average of three repeated recordings of material, each derived from pooled samples of 30 PMs.

### 4.7. Thin Layer Chromatography

It was necessary to remove the unreacted BODIPY fluorescent label prior to chromatography of the labelled disaccharides. The samples were purified as follows: water (5 μL) and n-butanol (5 μL) were added to the dried, labeled samples and applied to a silica-backed thin layer chromatography plate and subjected to 3 consecutive ascents in n-butanol. The labeled sugars remained low on the plate, while the unreacted label migrated close to the solvent front. The plate was dried and the silica removed from the first third of the plate (from Rf 0 to ca. 0.33) and collected, then extracted 3 times (0.33 mL H_2_O); these three extracts were pooled, filtered twice through 0.2 μm filters, and applied to HPAEC analysis. 

### 4.8. High Performance Anion Exchange Chromatography

High performance anion exchange chromatography (HPAEC) was performed on each of the pooled labeled disaccharides as follows: 1 mL of the pooled samples in 150 mM NaOH (aq.) was injected onto a ProPac PA-1 analytical column (4 × 250 mm, ThermoFisher Scientific, Loughborough, UK) pre-equilibrated in 150 mM NaOH (aq.). The column was held under isocratic flow (150 mM NaOH (aq.)) for 11 min prior to developing a linear gradient from 0 to 1 M NaCl (in isocratic 150 mM NaOH (aq.)) over 30 min. Standards, comprising the BODIPY-labeled 8 common disaccharides found in heparin and heparan sulphate (Figure 3), were also run under identical conditions prior to the samples and afterwards. 

## Figures and Tables

**Table 1:** Summary of heparan sulphate proteoglycan (Syndecan-1) and HS biosynthetic enzymes (EXT1, NDST-1, 2OST-1, 6OST-1, and 3OST-1) and their percentage identities to mammalian forms that were identified in the genome of *Glossina morsitans morsitans* following sequence searches in Vector base. Uniprot accession numbers of the mammalian proteins and expressed sequence tag entries (ESTs) are also shown.

Protein (Uniprot Accession Number)	EST Entry	E-Value	Identity (%)
Syndecan-1 (P18827)	EZ422907.1	5 × 10^−9^	52.2
EXT1 (Q16394)	FM983148.1	4 × 10^−48^	46
NDST-1 (P52848)	FM959941.1	2 × 10^−17^	29.2
2OST-1 (Q7LGA3)	DV604335.1	2 × 10^−73^	55.7
6OST-1 (O60243)	FM985074.1	1 × 10^−36^	67.4
3OST-1 (O14792)	FM959941.1	1 × 10^−54^	40.1

## References

[1] Service M. (2012). Medical Entomology.

[2] Peacock L., Ferris V., Sharma V., Sunter J., Bailey M., Carrington M., Gibson W. (2011). Identification of the meiotic life cycle stage of *Trypanosoma brucei* in the tsetse fly. Proc. Natl. Acad. Sci. USA.

[3] Miller N., Lehane M.J. (1993). Ionic environment and the permeability properties of the peritrophic membrane of *Glossina morsitans morsitans*. J. Insect Physiol..

[4] Rose C., Belomonte R., Armstrong S.D., Molyneux G., Haines L.R., Lehane M.J., Wastling J., Acosta-Serrano A. (2014). An Investigation into the Protein Composition of the Teneral *Glossina morsitans morsitans* Peritrophic Matrix. PLoS Negl. Trop. Dis..

[5] Lehane M.J., Allingham P.G., Weglicki P. (1996). Composition of the peritrophic matrix of the tsetse fly, *Glossina morsitans morsitans*. Cell Tissue Res..

[6] Abubakar L.U., Bulimo W.D., Mulaa F.J., Osir E.O. (2006). Molecular characterization of a tsetse fly mid-gut proteolytic lectin that mediates differentiation of African trypanosomes. Insect Biochem. Mol. Biol..

[7] Hao Z., Kasumba I., Lehane M.J., Gibson W.C., Kwon J., Aksoy S. (2001). tsetse immune responses and trypanosome transmission: Implications for the development of tsetse-based strategies to reduce trypa-nosomiasis. Proc. Natl. Acad. Sci. USA.

[8] Weiss B.L., Wang J., Maltz M.A., Wu Y., Aksoy S. (2013). Trypanosome infection establishment in the tsetse fly gut is influenced by microbiome-regulated host immune barriers. PLoS Pathog..

[9] Weiss B.L., Savage A.F., Griffith B.C., Wu Y., Aksoy S. (2014). The peritrophic matrix mediates differential infection outcomes in the tsetse fly gut following challenge with commensal, pathogenic, and parasitic microbes. J. Immunol..

[10] Aksoy E., Vigneron A., Bing X., Zhao X., O’Neill M., Wu Y.N., Bangs J.D., Weiss B.L., Aksoy S. (2016). Mammalian African trypanosome VSG coat enhances tsetse’s vector competence. Proc. Natl. Acad. Sci. USA.

[11] International Glossina Genome Initiative (2014). Genome sequence of the tsetse fly (*Glossina morsitans*): Vector of African trypanosomiasis. Science.

[12] Calvet C.M., Toma L., De Souza F.R., Meirelles M.D.E., Pereira M.C. (2003). Heparan sulphate proteoglycans mediate the invasion of cardiomyocytes by Trypanosoma cruzi. J. Eukaryot. Microbiol..

[13] Rostand K.S., Esko J.D. (1997). Microbial adherence to and invasion through proteoglycans. Infect. Immun..

[14] Dale C., Maudlin I. (1999). *Sodalis gen. nov*. and *Sodalis glossinidius* sp. *nov.* a microaerophilic secondary endosymbiont of the tsetse fly *Glossina morsitans morsitans*. Int. J. Syst. Bacteriol..

[15] Haines L.R., Lehane S.M., Pearson T.W., Lehane M.J. (2010). Tsetse EP protein protects the fly midgut from trypanosome establishment. PLoS Pathog..

[16] Kamimura K., Maeda N., Nakato H. (2011). In vivo manipulation of heparan sulphate structure and its effect on *Drosophila* development. Glycobiology.

[17] Bax X., Van der Schueren B., Cassiman J.J., Van der Berghe H., David G. (1994). Differential expression of multiple cell-surface heparan sulphate proteoglycans during embryonic tooth development. J. Histochem. Cytochem..

[18] David G., Bai X.M., Van der Schueren B., Cassiman J.J., Van den Berghe H. (1992). Developmental chang-es in heparan sulphate expression: In situ detection with mAbs. J. Cell Biol..

[19] Desai U.R., Wang H.-M., Linhardt R.J. (1993). Specificity studies on the heparin lyases from *Flavobacterium heparinium*. Biochemistry.

[20] Skidmore M.A., Guimond S.E., Dumax-Vorzet A.F., Atrih A., Yates E.A., Turnbull J.E. (2006). High sensitivity separation and detection of heparan sulphate disaccharides. J. Chromatogr. A.

[21] Powell A.K., Yates E.A., Fernig D.G., Turnbull J.E. (2004). Interactions of heparin/heparan sulphate with pro-teins: Appraisal of structural factors and experimental approaches. Glycobiology.

[22] Perrimon N., Bernfield M. (2000). Specificities of HS proteoglycans in developmental processes. Nature.

[23] Kusche-Gullberg M., Nybakken K., Perrimon N., Lindahl U. (2012). *Drosophila* heparan sulphate, a novel design. J. Biol. Chem..

[24] Chen X., Li S., Li C., Zhao S. (1999). Phylogeny of genus *Glossina* (*Diptera*: *Glossinidae*) according to ITS2 sequences. Sci. China C.

[25] Ford J. (1970). The Geographical Distribution of Glossina.

[26] Bai X., Bame K.J., Habuchi H., Kimata K., Esko J.D. (1997). Turnover of heparan sulphate depends on 2-*O*-sulphation of uronic acids. J. Biol. Chem..

[27] Dinglasan R.R., Alaganan A., Ghosh A.K., Saito A., van Kuppevelt T.H., Jacobs-Lorena M. (2007). *Plasmodium falciparum* ookinetes require mosquito midgut chondroitin sulphate proteoglycans for cell invasion. Proc. Natl. Acad. Sci. USA.

[28] Altschul S.F., Madden T.L., Schäffer A.A., Zhang J., Zhang Z., Miller W., Lipman D.J. (1997). Gapped BLAST and PSI-BLAST: A new generation of protein database search programs. Nucleic Acids Res..

[29] Giraldo-Calderón G.I., Emrich S.J., MacCallum J.M., Maslen G., Dialynas E., Topalis P., Ho N., Gesing S., Collins F.H., Lawson D. (2015). VectorBase: An updated bioinformatics resource for invertebrate vectors and other organisms related with human diseases. Nucleic Acids Res..

